# Supplementary material for: Immunogenicity and innate immunity to high-dose and repeated vaccination of modified mRNA versus unmodified mRNA
Source: Mol Ther Nucleic Acids. 2025 Jun 9;36(3):102588. doi: 10.1016/j.omtn.2025.102588 (PMC12221724; doi:10.1016/j.omtn.2025.102588)
Supplement: Document S1. Figures S1–S5 and Tables S1 and S2 [file mmc1.pdf]

OMTN, Volume *36*

Supplemental information

Immunogenicity and innate immunity to high-dose and repeated vaccination of modified mRNA versus unmodified mRNA

Olivia Engstrand, Gustav Joas, Marcos C. Miranda, Xianglei Yan, Klara Lenart, Rodrigo Arcoverde Cerveira, Annika Reinhardt, and Karin Loré

SUPPLEMENTAL MATERIALS

SUPPLEMENTAL FIGURES

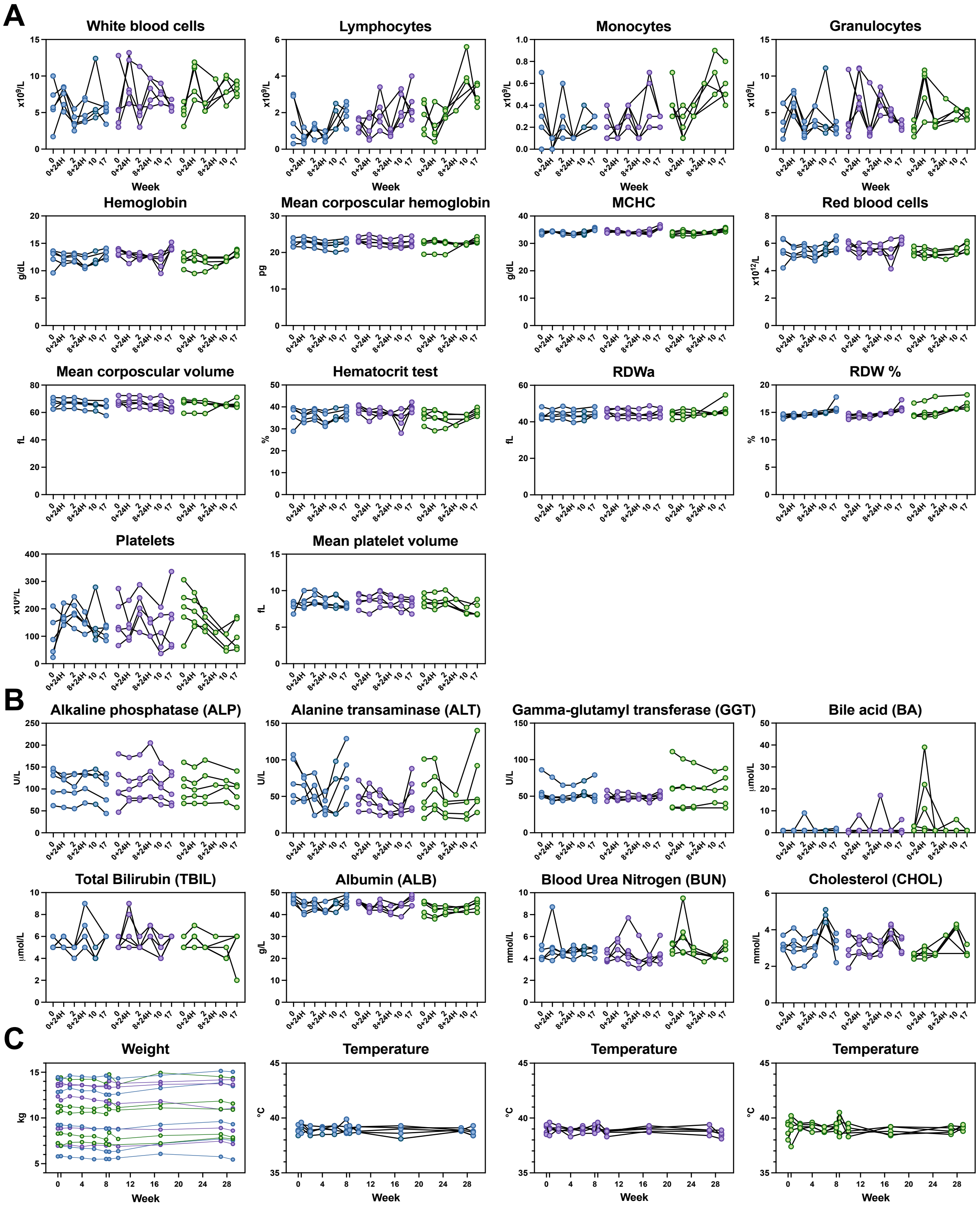

Figure S1: Safety data after repetitive immunizations. Unmodified mRNA 160µg (blue), Modified mRNA 400µg (purple) and Modified mRNA 800µg (green). (A) Hematological paramaters and (B) Clinical chemistry at baseline, 24 hours and 2 weeks post first immunization and 24 hours, 2 weeks and 9 weeks post fifth immunization. (C) Weight and temperature at each sampling time point.

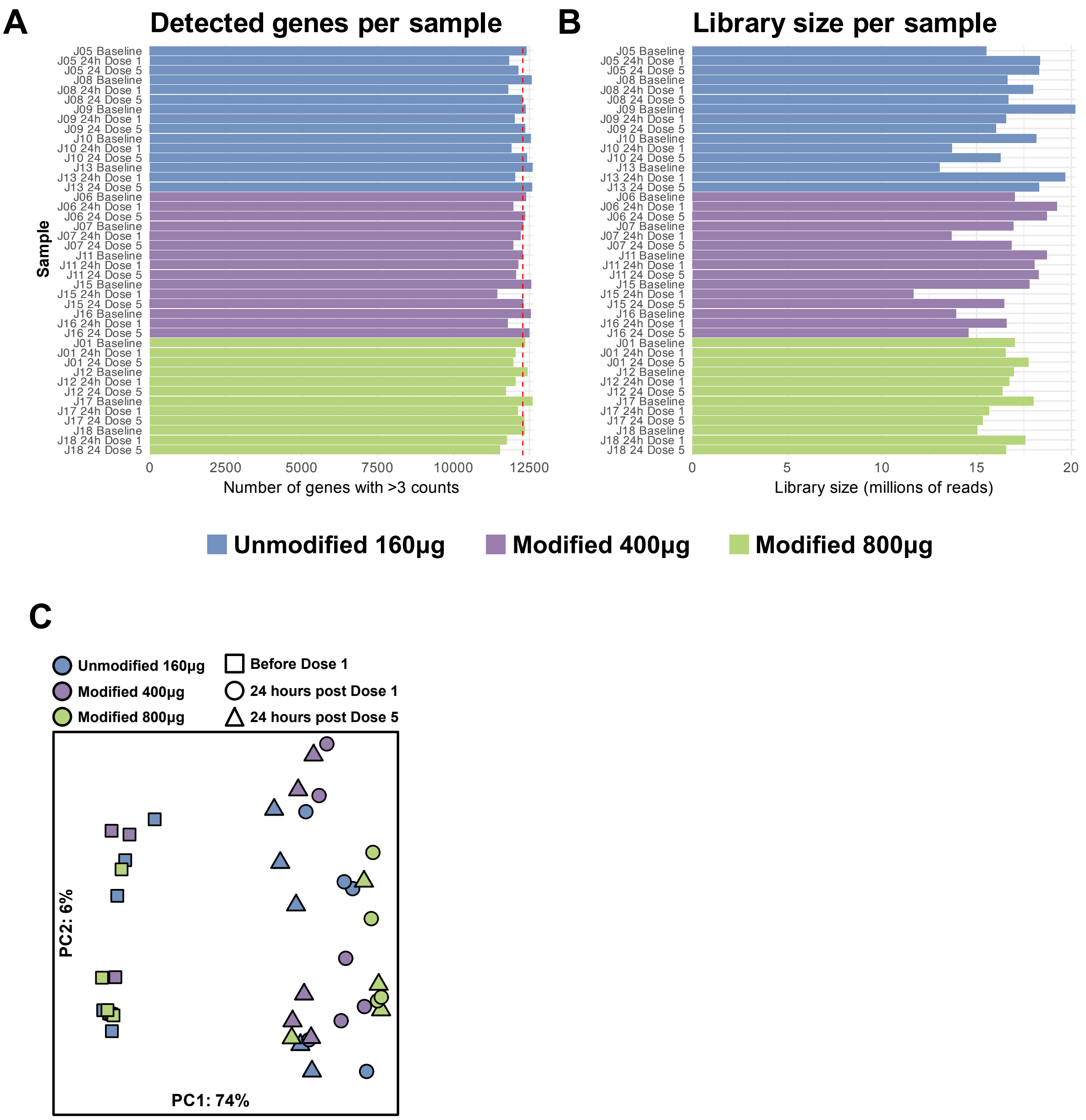

Figure S2: RNA sequencing quality control (A) Bars show the number of genes with >3 raw counts in each RNA-seq sample. The dashed red line indicates the median number of detected genes across all samples. (B) Total read counts (in millions) for each sample prior to normalization. (C) Principal Component Analysis colored by group and shaped by sampling time point.

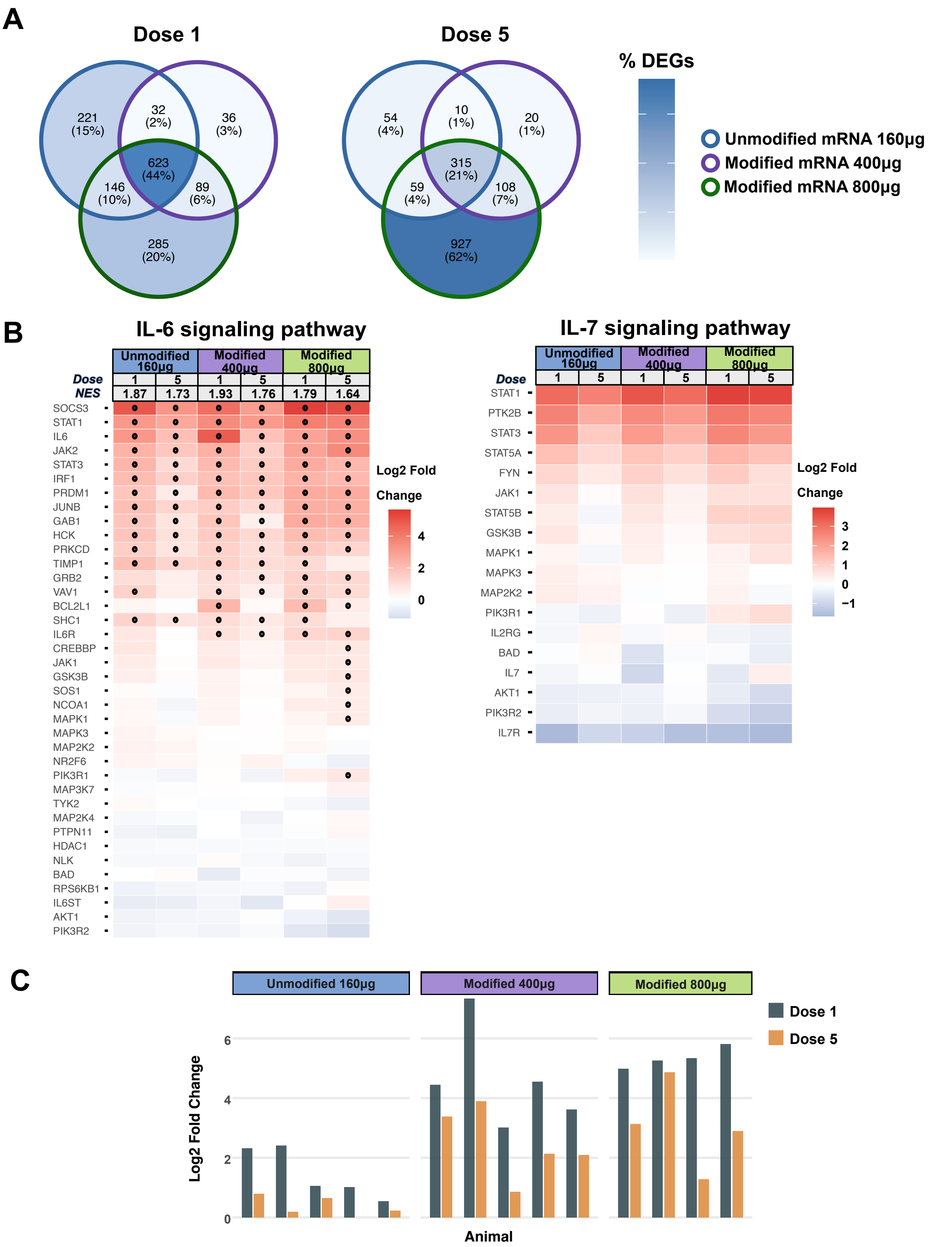

Figure S3: Transcriptomic Changes Across Vaccine Groups and Timepoints (A) Venn diagrams illustrating the percentage of overlapping DEGs across groups and doses. Exclusive regions represent DEGs unique to each group. Shared regions, including the central intersection, show DEGs common to two or more groups. (B) Heatmaps of $\log_2$ fold changes ($\log_2$FC) for genes in the IL-6 and IL-7 signaling pathways, as defined in the WikiPathways database, across groups and timepoints. Black circles indicate core enriched genes as identified by Gene Set Enrichment Analysis. (C) Barplot showing *gag* expression levels detected by RNA-seq 24 hours after vaccination, stratified by vaccine group and dose. gag was undetectable at baseline. The negative control gene, env (not shown), was not detected in any sample. NES = normalized enrichment score.

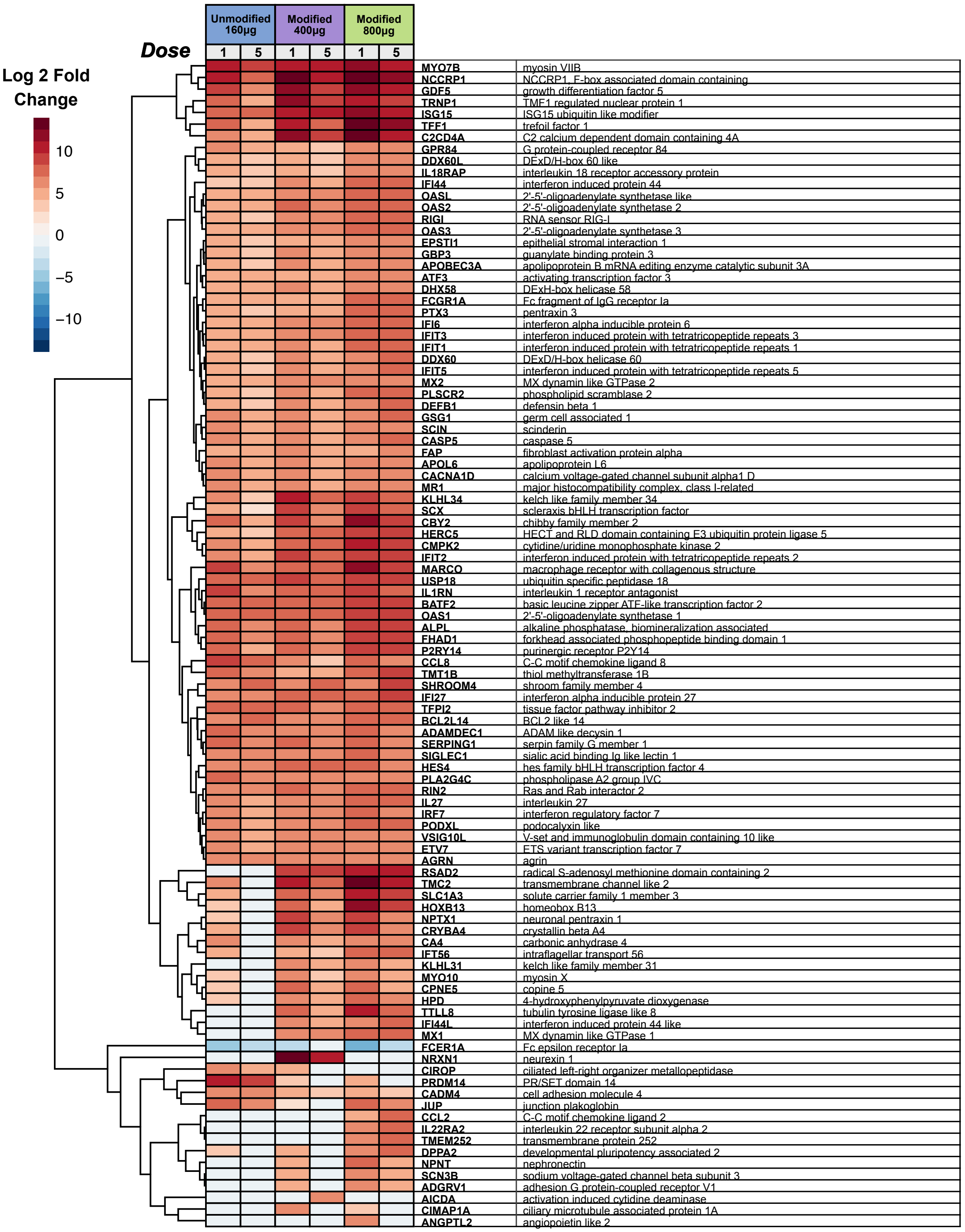

Figure S4: Top 100 differentially expressed genes (DEGs) across groups and timepoints following vaccination. DEGs with the highest absolute $\log_2$ fold changes and False Discovery Rate adjusted *p*-values < 0.05 across all groups and timepoints. Comparing baseline (0 hours, pre-immunization) to 24 hours post-immunization following the first and fifth doses. Gene descriptions are shown on the right. Genes were hierarchically clustered using Ward's method. Missing values were imputed as 0.

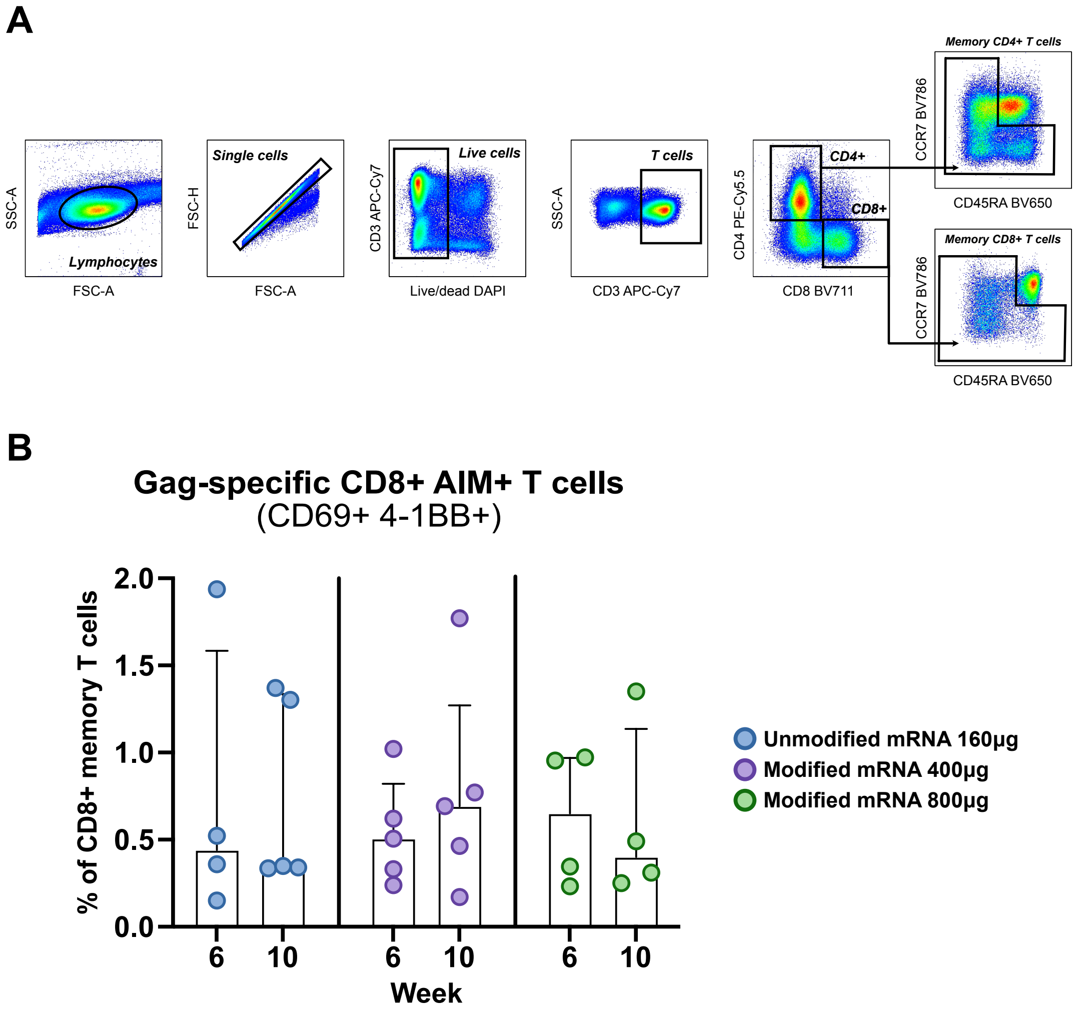

Figure S5: Gag-specific T cell responses. (A) Representative gating strategy to identify memory T cell subsets. (B) Frequency of Gag-specific CD8+ CD69+ 4-1BB+ T cells analyzed from antigen recall assay with fresh PBMCs. Graph is showing background-subtracted frequencies calculated based on corresponding DMSO-control, Data presented as group medians (IQR).

SUPPLEMENTAL TABLES

Table S1: Immunophenotyping antibody panel.

Fluorochrome	Marker	Clone	Company	Cat. no.
FITC	CD40	5C3	BioLegend	334306
PE	NKg2a (CD159a)	Z199	Beckman Coulter	IM3291U
BV421	CD80	L307.4	BD	564160
PE-Dazzle 594	CCR7	G043H7	Biolegend	353236
PerCp-Cy5.5	CD123	7G3	BD	558714
APC-Cy7	CD3	SP34-2	BD	557757
APC	CD66	TET2	Miltenyi	130-118-539
BV786	CD70	Ki-24	BD	565338
BV650	HLA-DR	L243	Biolegend	307650
PE-Cy7	CD11c	3.9	BioLegend	301608
AF700	CD16	38G	BD	560713
BV605	CD20	2H7	BioLegend	302334
BV510	CD14	M5E2	Biolegend	301842
DAPI (AF350)	Live Dead Fixable Blue	-	Life Tech	L-23105

Table S2: T cell assay antibody panel

Fluorochrome	Marker	Clone	Company	Cat. no.
CD107a	BV421	H4A3	Biolegend	328626
OX40	BV510	L106	BD	745040
CCR7	BV786	G043H7	Biolegend	353230
CD8a	BV711	RPA-T8	Biolegend	301044
CD4	PE-Cy55	S3.5	Invitrogen	MHCD0418
CD45RA	BV650	5H9	BD	740608
4-1BB	APC	4B4-1	BD	550890
IL-2	BV605	MQ1-17H12•	BD	564165
CD69	ECD	TP1.55.3	Beckman	6607110
CD3	APC-Cy7	SP34.2	BD	557757
IFNg	AF700	B27	Biolegend	506516
DAPI (AF350)	Live Dead Fixable Blue	-	Life Tech	L-23105
