# Supplementary material for: Immunogenicity and innate immunity to high-dose and repeated vaccination of modified mRNA versus unmodified mRNA
Source: Mol Ther Nucleic Acids. 2025 Jun 9;36(3):102588. doi: 10.1016/j.omtn.2025.102588 (PMC12221724; doi:10.1016/j.omtn.2025.102588)
Supplement: Document S2. Article plus supplemental information [file mmc2.pdf]

Molecular Therapy
Nucleic Acids
Brief report

Immunogenicity and innate immunity to high-dose and repeated vaccination of modified mRNA versus unmodified mRNA

Olivia Engstrand,[1,2] Gustav Joas,[1,2] Marcos C. Miranda,[1,2] Xianglei Yan,[1,2] Klara Lenart,[1,2] Rodrigo Arcoverde Cerveira,[1,2] Annika Reinhardt,[1,2] and Karin Loré[1,2]

[1]Division of Immunology and Respiratory Medicine, Department of Medicine Solna, Karolinska Institutet and Karolinska University Hospital, 171 76 Stockholm, Sweden; [2]Center for Molecular Medicine, 171 64 Stockholm, Sweden

mRNA vaccines represent a new era with several novel constructs underway. We compared the responses of high doses and multiple repetitive immunizations of a nucleoside-modified mRNA construct to a sequence-codon-optimized unmodified mRNA construct encoding the identical model antigen (HIV-1 gag). Rhesus macaques were immunized five times at 2-week intervals, with a final boost 20 weeks later. At 24 h post-vaccination, both unmodified (160 μg) and modified (400 μg and 800 μg) mRNA constructs elicited clear but transient increase of plasmacytoid dendritic cells, intermediate CD14+ CD16+ monocytes, and neutrophils along with secretion of type I interferon (IFN)-related and inflammatory cytokines. Unmodified mRNA induced higher interleukin-7 (IL-7) and IFN-α levels, whereas modified mRNA induced higher IL-6 levels. Transcriptomic profiling showed significant upregulation of genes related to type I IFN signaling, antigen presentation, and innate immune activation induced by both mRNA constructs. The high-dose modified mRNA induced a higher number of differentially expressed genes at prime, which further increased after the fifth immunization. These differences in innate immune activation nonetheless led to similar levels and kinetics of gag-specific antibody and T cell responses. These findings offer insights into the immunogenic and reactogenic potential of different mRNA vaccine modalities, guiding future vaccine and therapy development.

INTRODUCTION

While the first mRNA vaccine tested in humans in 2013 was based on sequence-codon optimized unmodified mRNA,[1] the currently licensed vaccines are based on N1-methylpseudouridine-modified mRNA (e.g., BNT162b2; BioNTech/Pfizer, mRNA-1273; Moderna, mRNA-1345; Moderna). Due to a higher incidence of adverse reactions,[2] likely caused by strong innate immune activation,[3–5] lower doses of unmodified mRNA vaccines appear necessary compared to modified mRNA vaccines. Nevertheless, sequence-engineered unmodified mRNA has shown good immunogenicity[6,7] and may be applicable as therapeutics for certain diseases such as for cancer treatment. Indeed, there are multiple ongoing human trials assessing the clinical utility of therapeutic cancer vaccines based on either unmodified mRNA (NCT05938387; NCT00923312[8]) or modified mRNA (NCT02410733,[9] NCT05933577, NCT03897881, NCT03313778). Therapeutic cancer vaccines typically require higher mRNA doses and more frequent administrations compared to prophylactic mRNA vaccines to infectious diseases. Repetitive high-dose modified mRNA immunizations recently showed anti-tumor activity in combination therapy.[10–12]

The purpose of the current study was to generate immunological data to aid in the development of therapeutic cancer vaccines based on the mRNA technology. We compared both the early innate immune activation as well as the adaptive responses to high doses and multiple immunizations of a modified mRNA versus an unmodified mRNA construct encoding the identical HIV-1 gag antigen in rhesus macaques. The gag antigen was solely used as a model antigen since it has been frequently used in macaques.[11,12] In addition, the study design was specifically chosen to explore limits of mRNA vaccine reactogenicity, innate immune activation, and adaptive immune responses.

RESULTS

Rapid and strong but transient immune cell fluctuation and cytokine secretion in all groups

Rhesus macaques were divided into groups with five animals per group (Figure 1A). They were immunized with unmodified (160 μg) or N1-methylpseudouridine-modified (400 μg and 800 μg) mRNA vaccine constructs five times at 2-week intervals, with a final boost 20 weeks later. This approach was chosen to assess the impact on innate immune activation as well as T cell and antibody responses. Similar to a clinical setting, the unmodified mRNA construct was given at lower dose than the modified

Received 23 January 2025; accepted 5 June 2025;
https://doi.org/10.1016/j.omtn.2025.102588.

Correspondence: Karin Loré, Division of Immunology and Respiratory Medicine, Department of Medicine Solna, Karolinska Institutet and Karolinska University Hospital, 171 76 Stockholm, Sweden.
E-mail: karin.lore@ki.se

© 2025 The Author(s). Published by Elsevier Inc. on behalf of The American Society of Gene and Cell Therapy. This is an open access article under the CC BY license (http://creativecommons.org/licenses/by/4.0/).

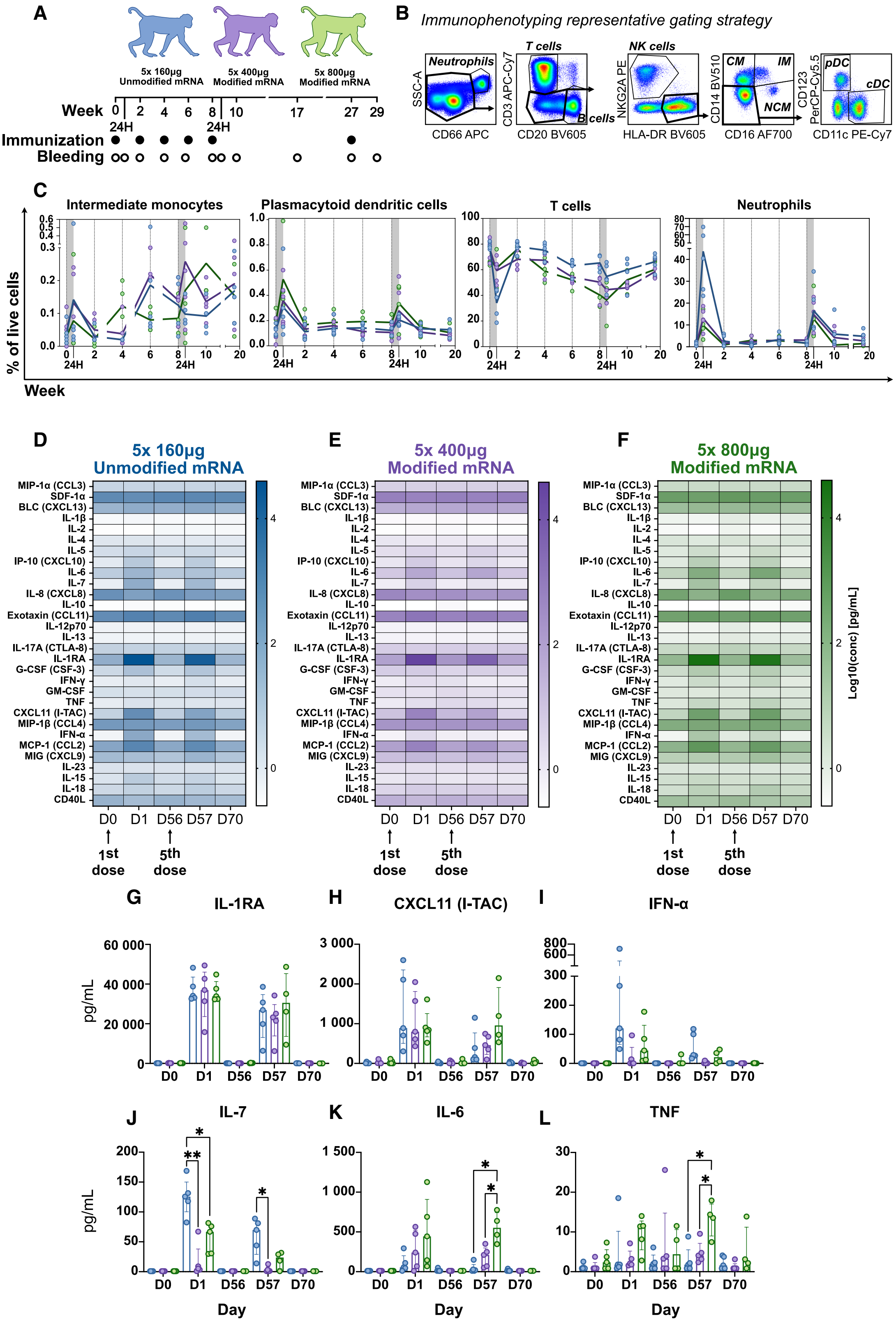

(legend on next page)

mRNA. The gag protein was selected as a model antigen since responses are well characterized in non-human primates (NHPs).[13,14]

At 24 h after immunization, safety monitoring parameters showed either no or minor transient fluctuations, majority within clinical chemistry and hematological reference intervals[15] (Figures S1A and S1B). Animals did not show any behavioral changes, increase in body temperature, or weight loss during the study (Figure S1C). However, it is noteworthy that the NHP model exhibits greater resistance to mRNA-vaccine-induced inflammation compared to humans.[16]

At 24 h post-vaccination, both unmodified and modified mRNA constructs elicited a transient increase of intermediate (inflammatory) CD14+ CD16+ monocytes (Figures 1B and 1C). This is in line with earlier reports by others and us of monocyte expansion following mRNA vaccination both in humans and NHPs.[17–21] Simultaneously, there was a notable increase in plasmacytoid dendritic cells and neutrophils, along with a transient decrease in T cells, which may suggest a temporary redistribution of lymphocytes to tissues and an increase in circulating myeloid cells (Figures 1B and 1C). We found a similar change in cell proportions after the prime immunization as with the fifth immunization.

At 24 h, there was also secretion of multiple cytokines and chemokines such as interleukin-1 receptor antagonist (IL-1RA) and type-I-IFN-associated CXCL11 and IFN-α (Figures 1D–1I). There was donor variability as expected,[19] but the levels were largely similar between the groups. All cytokines were induced to similar levels by the fifth immunization, although there was a trend toward lower levels of IL-7 and IFN-α in the groups receiving unmodified and high-dose modified mRNA vaccine. Overall, IL-7 and IFN-α tended to be induced at higher levels by the unmodified mRNA vaccine, whereas IL-6 was induced at higher levels in the groups receiving the modified mRNA vaccine (Figures 1J–1L). In addition, certain inflammatory cytokines, such as IL-6 and TNF, showed the highest levels in the high-dose group compared to the low dose of the modified mRNA vaccine (Figures 1K–1L).

Dose-dependent immune activation and gene expression modulation following multiple mRNA immunizations

Transcriptomic profiling revealed significant modulation in gene expression following both the first and fifth immunizations, with varying number of differentially expressed genes (DEGs) in the groups (Figures 2A and S2). In the unmodified mRNA and lower dose modified mRNA groups, the number of DEGs was higher after the first dose compared to the fifth. In contrast, the higher modified mRNA dose, which resulted in the largest number of DEGs after the first dose, showed a further increase after the fifth dose (Figure 2A). This may indicate a dose-dependent increase in immune activation and the potential of booster doses to amplify the response at this high mRNA concentration.

To further evaluate overall similarity in gene expression, we calculated Pearson correlation coefficients based on the log2 fold changes of all genes between the groups (Figures 2B and 2C). Transcriptional profiles were highly similar following the first dose ($r > 0.88$). By the fifth dose, correlations remained relatively strong ($r = 0.71–0.85$), although slightly reduced, reflecting the increased magnitude of transcriptional changes observed in the high-dose modified group. Notably, almost all DEGs identified in the two lower dose groups were also appearing in the high-dose group (Figure S3A). While the high-dose group exhibited a greater number of unique DEGs after the fifth dose, these additional DEGs tended to have lower fold changes. The differentially expressed genes with the largest log2 fold changes remained consistent across all groups. For a list of the top 100 DEGs and their corresponding expression heatmap across groups, see Figure S4.

Gene set enrichment analysis (GSEA) using defined blood transcription modules[22] revealed the induction of gene sets linked to type I IFN signaling, antigen presentation, and innate immune activation (Figure 2D), consistent with prior findings.[17,20] Overall, the gene set enrichment pattern was similar between the groups and between the first and fifth dose. To further investigate whether the differences in cytokine levels—specifically IL-6 and IL-7—were reflected at the transcriptomic level, we performed GSEA using the WikiPathways database.[23] This analysis revealed enrichment of the IL-6 signaling pathway across all groups and time points. In contrast, although IL-7 exhibited a similar fold change pattern across groups in the RNA sequencing (RNA-seq) data, it did not reach statistical significance in the enrichment analysis (Figure S3B).

From the mRNA transcriptomics data, we detected *gag* expression from the mRNA vaccines. The *gag* sequence was consistently detected 24 h after vaccination but absent pre-vaccination, indicating that the mRNA vaccine disseminated into the circulation (Figure S3C). We also included the HIV *env* gene sequence as a negative control, which was not detected in any sample. We observed lower levels of *gag* expression in the unmodified mRNA group, which may be attributed to differences between the codon-optimized sequence in the unmodified mRNA vaccine and the reference *gag* sequence used as the query in the transcriptomics data, making direct comparison of expression levels between the groups challenging.

Figure 1. Immune responses following immunization with unmodified or modified mRNA vaccine constructs

(A) Study design. (B) Gating strategy used in immune cell phenotyping. CM, classical monocytes; IM, intermediate monocytes; NCM, nonclassical monocytes; pDC, plasmacytoid dendritic cells; cDC, conventional dendritic cell. (C) Mean frequencies of immune cell populations in blood over time. (D–F) Cytokine and chemokine concentrations in plasma. (G–L) Comparison between vaccine groups of selected plasma cytokines after the first and fifth immunization; colored bars display medians (IQR) of each group. Statistical differences between groups were calculated with the mixed effects model with Geisser-Greenhouse correction and Tukey's multiple comparisons test; $^{*}p < 0.05$, $^{**}p < 0.01$, $^{***}p < 0.001$.

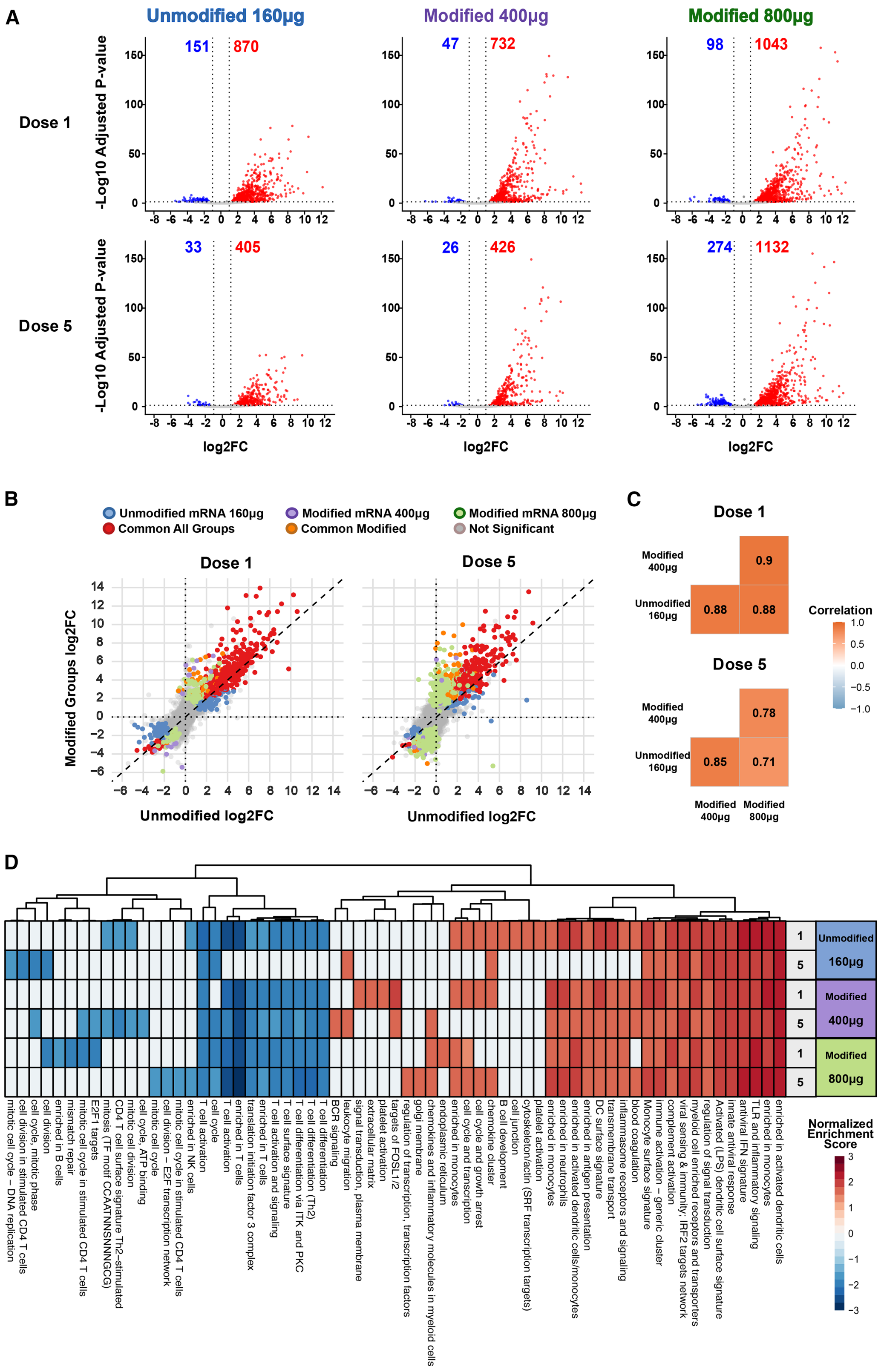

(legend on next page)

Furthermore, no significant difference was observed between the low- and high-dose modified mRNA groups, which suggests that the doses used in our study were saturating. Another notable observation was that the boost immunization resulted in lower gag expression compared to the prime immunization across all animals and groups. We speculate that the existing immunity, such as Gag-specific CD8+ T cells and antibodies, present at the time of the boost may partly eliminate Gag-producing cells that carry the mRNA vaccine.

Rapid induction of T cell and B cell responses

All groups showed induction of well-detectable gag-specific antibody responses 2 weeks after the second immunization with further increasing titers up until the fifth dose, followed by waning (Figure 3A). However, the final boost given with the longer interval enhanced the titers back to peak levels. Both the kinetics as well as the levels of antibodies were similar between the groups (Figures 3A and 3B).

Gag-specific memory T cell responses as measured by both intracellular cytokine staining and activation-induced marker expression (AIM) upon antigen recall assay were readily detected after three immunizations in all groups at similar frequencies (Figures 3C–3F and S5). Both gag-specific CD4+ and CD8+ memory T cell responses were induced by the vaccines as demonstrated by increased frequencies of 4-1BB + OX40+ and CD69+ IFN-γ-producing CD4+ T cells as well as CD69+ 4-1BB + or IFN-γ-producing CD8+ T cells, respectively. Consistent with previous studies showing rapid induction and dominance of CD4+ over CD8+ T cell responses upon mRNA vaccination in humans, gag-specific CD4+ T cell responses occurred at higher frequencies in all groups and were dominated by a CCR7− CD45RA-effector memory or CCR7+ CD45RA-central memory phenotype. Of note, CD4+ T cell responses did not significantly increase between the second and fifth immunization, and doubling the dose of modified mRNA did not generate higher numbers of gag-specific CD4+ T cell responses.

In conclusion, at this dosing, the modified and unmodified mRNA constructs induced similar adaptive responses.

DISCUSSION

The high doses used here enabled assessments of immune activity, potential reactogenicity, and saturation of immune responses. While the frequent dosing schedule aimed to provoke excessive immune activation, such regimens are being considered for therapeutic cancer vaccination (NCT05938387, NCT00923312, and NCT05933577). As tumor antigens are often poorly immunogenic, repetitive immunizations of the cancer vaccine with high doses and a strong adjuvant effect appear required to induce potent immune responses. In our study, fewer immunizations with longer intervals could plausibly have yielded similar peak antibody and T cell responses.

NHPs are valuable for assessing mRNA vaccines, though they tend to be more resistant to cytokine-driven side effects than humans.[16] Although mice and NHPs were shown to respond with higher production of IFN-α and IL-1RA, the overall innate immune activation pattern is similar across species.[17,24,25] Both vaccine constructs tested in this study led to an increase in intermediate monocytes and secretion of type-I-IFN-related and inflammatory cytokines. We have earlier found that a clinical dose (8 μg) of the unmodified COVID-19 vaccine candidate CVnCoV showed undetectable TNF and IL-6[18] in contrast to the high-dose unmodified mRNA (160 μg) used in this study, demonstrating dose-dependent inflammation.

The fact that unmodified mRNA induced more IL-7 and IFN-α while modified mRNA induced more IL-6 indicates some differences in innate immune responses. The trend toward slightly higher levels of IFN-α in animals immunized with unmodified mRNA is consistent with stronger Toll-like receptor (TLR), inflammatory, and antiviral IFN signaling as identified on the RNA level, indicative of more TLR7/8 activation in this group. This is in line with previous studies demonstrating activation of distinct TLR signaling pathways for unmodified and modified mRNA vaccines.[9,26] However, it is important to note that both mRNA constructs were capable of inducing the same cytokines, and the RNA transcriptomics data showed that genes of the IL-6 pathway, for example, were highly upregulated in all groups.

Nevertheless, nuances in the innate immune responses between unmodified and modified mRNA may have downstream consequences for polarizing adaptive immunity. In the context of vaccination, IL-7 has been discussed as molecular adjuvant improving vaccine responses by enhancing both T cell and humoral immune responses.[27] Increased presence of IL-7 could thus be potentially beneficial for therapeutic cancer vaccines. Indeed, clinical trials subcutaneously administering IL-7, together with a cancer vaccine, demonstrated an increase in T cell responses in combination therapy (NCT01881867[28] and NCT00923351), though more studies are needed for further evaluation.[29]

On another note, previous studies have further shown that IL-6 is induced by lipid nanoparticles (LNPs), which contributes to an adjuvant effect of mRNA vaccines.[30] Since the modified mRNA vaccine was

Figure 2. Unmodified vs. modified mRNA transcriptional profile in blood post-immunization

(A) Volcano plots displaying fold changes and *p* values calculated by Wald test between baseline (0 h, pre-immunization) and 24 h post-immunization after first and fifth dose. Each plot shows total number of differentially expressed genes, cut-off absolute log2 fold change (log_2FC) > 1, FDR-adjusted $p < 0.05$. (B) Scatterplots of log_2FC in gene expression between the unmodified mRNA vaccine group (160 μg) and the modified mRNA vaccine groups (400 μg and 800 μg), at dose 1 and dose 5. Each point represents a gene; diagonal dashed lines indicate the identity line (x = y). Colors indicate differential expression classification across groups. (C) Pearson correlation coefficients of log_2FC values between vaccine groups at each dose, based on all genes. $p < 0.01$. (D) Gene set enrichment analysis based on fold change rankings 24 h post-vaccination after first and fifth dose using previously described blood transcription modules.[22] Gene modules colored based on absolute normalized enrichment score.

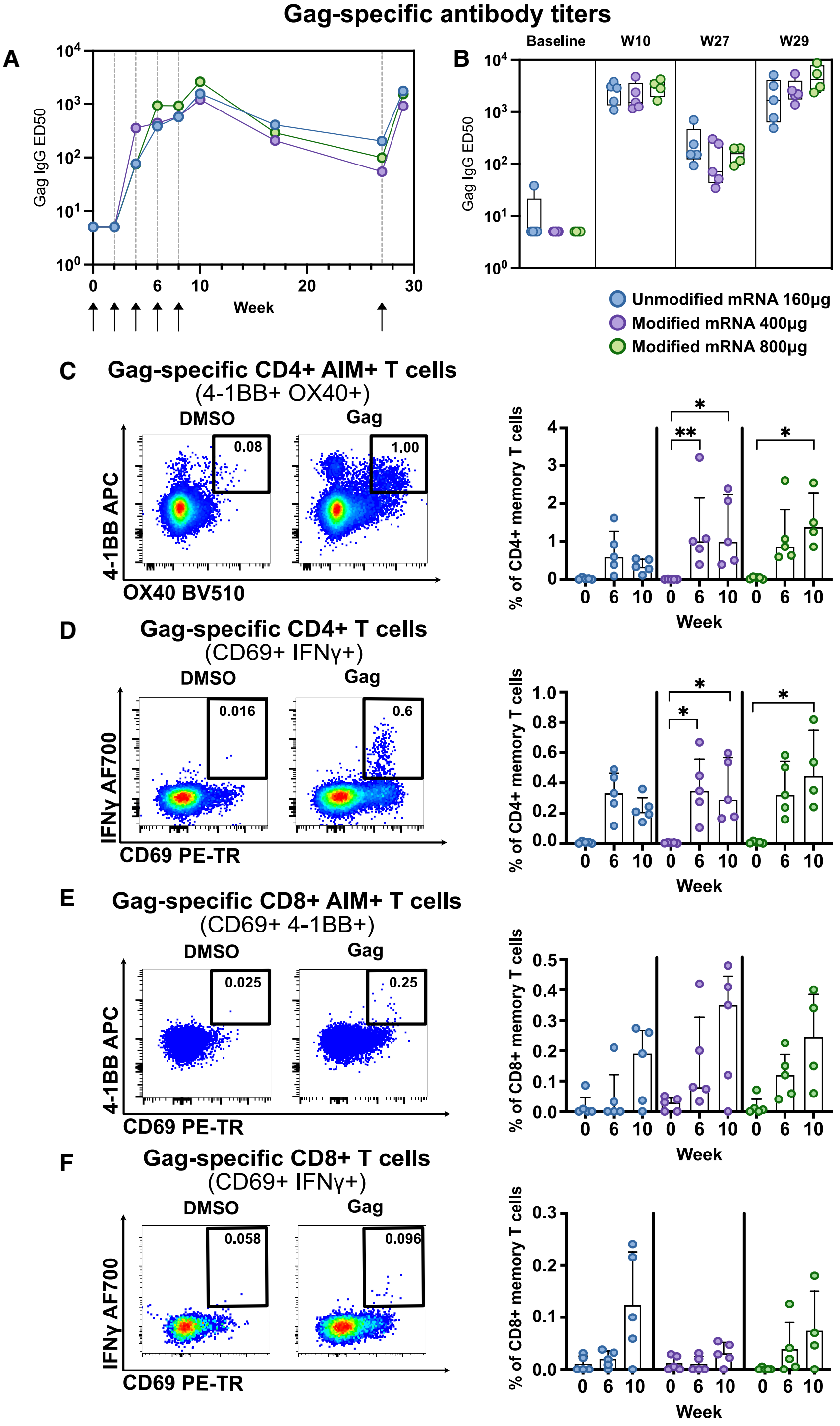

Figure 3. Both mRNA vaccine constructs induce gag-specific immune responses

(A and B) Plasma antibody IgG titers to HIV-1 gag p24, displayed with half-maximal effective dilution (ED50) measured by ELISA. (A) Gag-specific titers of pooled data per group over the study period. (B) Box and whisker plot of individual gag-binding titers at baseline, 2 weeks after fifth immunization, day of boost, and 2 weeks post-boost. Box lines indicate group median, and whiskers show minimum to maximum values. (C–F) Frequencies of gag-specific memory T cells analyzed from cryopreserved PBMCs at baseline, 2-weeks after third (W6) and fifth immunization (W10), displayed as medians (IQR) and paired with respective gating strategy. Background subtraction was calculated for all samples based on corresponding DMSO control, and statistical differences between time points were calculated with Kruskal-Wallis test and Dunn's test as post-hoc analysis; *$p < 0.05$, **$p < 0.01$, ***$p < 0.001$.

given at higher doses in our study, it is plausible that the higher amount of LNPs contributed to the higher IL-6 observed in these groups.

It has been proposed that mRNA vaccines can mediate trained immunity, i.e., epigenetic alterations to innate immune cell populations causing increased responsiveness to stimuli.[31] However, while Yamaguchi et al. reported short-term epigenetic modulation following mRNA vaccination, we[17] and others[17,32] did not find any functional or epigenomic changes. In the current study, we mostly observed similar cell activation, cytokine secretion, and RNA expression modulation after the prime immunization as with the fifth immunization using a 2-week interval schedule. Therefore, enhanced responsiveness due to trained immunity does not appear to be a major influencing factor. However, the high-dose modified mRNA group showed some enhanced upregulation after the fifth dose, which may be a result of excessive immune activation.

On the other hand, it is tempting to speculate that the repetitive immunizations could have induced tolerance in the innate immune compartment. We observed a slight trend toward lower levels of IL-7 and IFN-α in the groups receiving unmodified and high-dose modified mRNA vaccine, which may suggest potential induction of TLR tolerance. However, overall changes in cytokine, chemokine expression as well as cell activation between the different time points were minimal, indicating that TLR tolerance was not induced to a significant extent in this study.

Importantly, any differences in gene modulation and cytokine secretion between the different study groups did not translate into significant variations in T cell or antibody responses. Since doubling the dose of modified mRNA increased innate immune activation without enhancing gag-specific antibodies or T cells, the responses may

be saturated. This may also be due to a similar antigen load that did not increase with high doses of the mRNA vaccine, as our gag expression data showed similar levels between the low- and high-dose groups.

Unmodified mRNA vaccines are typically used at lower doses than modified mRNA in both preclinical and clinical studies due to reactogenicity concerns. A range of similar doses would have been ideal for direct comparison. Given the high cost and ethical considerations of NHP studies, we had to prioritize the study groups. While we recognize the limitations this may introduce, we believe that the chosen doses are appropriate for assessing innate and adaptive immunity, especially considering the broader context of cancer vaccine research.

Although it is well known that unmodified mRNA induces more innate immune activity and reactogenicity than modified, the very high doses used here likely masked some of these differences. Still, unmodified RNA showed as high immune activity at less than half the dose of modified. The choice of construct may ultimately depend on target disease, tolerability and production cost and scalability. While modified mRNA vaccines are generally preferred due to concerns over reactogenicity, an unmodified mRNA vaccine may be considered in situations where a simpler, more cost-effective production approach is required for high-dose regimens or where higher reactogenicity is acceptable for the target disease. Additionally, the data from the unmodified mRNA groups could have implications for self-amplifying RNA vaccines where the only clinically approved vaccine to date is based on unmodified mRNA.[33,34]

MATERIALS AND METHODS

Vaccines

Unmodified and pseudouridine-modified mRNA constructs encoding the HIV-1 gag p24 protein, designed and produced by CureVac SE, were encapsulated in LNPs.[14,35] The LNPs were the same for all mRNA constructs. Sequence optimization of CureVac's unmodified mRNA construct has previously been described (European patents EP1392341A2, EP1857122, application WO2012019780A1).[6,36,37]

Study design

Fifteen Indian rhesus macaques (*Macaca mulatta*, 7–10 years old) were housed in the Astrid Fagraeus Laboratory at Karolinska Institutet, Stockholm. All animal experiments adhered to the guidelines of the AAALAC and the Swedish Animal Welfare Agency, with approval from the Regional Animal Ethics Committee of Northern Stockholm. The animals received six intramuscular injections of mRNA vaccines (160–800 μg) and were sampled for 29 weeks as depicted in Figure 1A. Vaccine safety was assessed with hematological and biochemical parameters performed by Scantox (Solna, Sweden).

Sample processing

PBMCs were isolated by standard gradient density centrifugation from heparinized blood as described previously.[18]

Phenotyping

Immune cell subsets were monitored by flow cytometry over a 20-week time course, with a 24-h time point after the first and fifth dose. Isolated PBMCs were stained with an immunophenotyping panel (Table S1) as described previously,[18] acquired on BD LSRFortessa cell analyzer and analyzed with FlowJo v10.10.0.

Plasma cytokine and chemokine quantification

ProcartaPlex NHP Cytokine & Chemokine Panel 30plex (Thermo Fisher) was used according to manufacturer's instructions and analyzed using a MagPix (Luminex) as previously described.[17,18,38]

RNA sequencing and bioinformatic analysis

Blood samples were collected and processed for RNA extraction and sequencing as previously described[17,38] with an average sequencing depth of 50 M reads per sample. Raw reads were processed using the nf-core/rnaseq pipeline v3.14.0,[39] aligning to the *Macaca mulatta* genome build Mmul_10. To assess *gag* expression levels, spike-in sequences for *gag* and *env* were added to the pipelines genome index using the reference sequences from the NCBI RefSeq assembly (GCF_000864765.1).

Gene-level abundance estimates were generated from transcript-level quantification using the tximport package v1.32.0.[40] Gene annotations for *Macaca mulatta* were retrieved from Ensembl release 112 using the supporting package ensembldb v2.28.1.[41]

Lowly expressed genes with fewer than five raw counts in at least three samples were removed, retaining 13,488 genes out of an initial 21,738 for downstream analysis. Differential expression analysis was done with DESeq2 v1.44.0.[42] Genes were considered differentially expressed if they exhibited an absolute $\log_2$ fold change >1 and a false discovery rate adjusted p value <0.05.

GSEA was performed with clusterProfiler v4.12.6.[43] All post-filtered genes were used as the background universe. For pathway-specific analysis of IL-6 and IL-7 signaling, gene sets from the WikiPathways[23] database were used. Because pathway annotations are based on *Homo sapiens*, *Macaca mulatta* gene identifiers were converted to their human orthologs using the orthogene package v1.10.0 (https://doi.org/10.18129/B9.bioc.orthogene).

ELISA for antibodies

Plates were coated with recombinant HIV-1 Gag p24 (Bio-Techne) at 1 μg/mL, and ELISA was performed as previously described[18] to assess IgG titers in plasma.

Antigen-specific T cells

PBMCs were stimulated with 2 μg/mL Gag peptide pool (Peptides & Elephants) overnight. T cell assay was performed as described previously[44] using cryopreserved or fresh PBMCs and stained with a selected panel of intracellular and surface markers (Table S2).

Statistics

Statistical differences between groups in Figure 1 were calculated with the mixed effects model with Geisser-Greenhouse correction and Tukey's multiple comparisons test. Kruskal-Wallis test and Dunn's post-hoc test were applied to assess statistical differences between groups and time points in Figure 3. Non-parametric tests were used due to low sample size, where normal distribution could not be assumed. The results were considered statistically significant when $p < 0.05$; analysis was done in Graphpad Prism10 and R programming (v4.4.1).

DATA AVAILABILITY

RNA-seq raw data are available at European Nucleotide Archive (ENA): under the accession number PRJEB89689 and processed data together at Zenodo: https://doi.org/10.5281/zenodo.14609902. Code is available at github.com/Lore-Lab-Vaccine-Immunology/modified_unmodified_mrna; commit ID: a826133.

ACKNOWLEDGMENTS

We are grateful to CureVac SE for financial support, provision of mRNA constructs, and assistance with study design. We acknowledge the team from Affinity Proteomics-Stockholm at SciLifeLab Sweden for technical support and the generation of systemic cytokine data for this project. We would like to thank BEA, the Bioinformatics and Expression Analysis core facility, which is supported by the board of research at the Karolinska Institutet. This work was supported by grants from the Swedish Research Council (2019-01036 to K.Loré.). Also, this research was supported by intramural faculty salary grants from Karolinska Institutet (K.Lenart.) and a grant from the China Scholarship Council (X.Y.).

AUTHOR CONTRIBUTIONS

Conceptualization, O.E., M.M., A.R., and K.Loré.; formal analysis, O.E., M.M., G.J., R.A.C., A.R., and K.Loré.; funding acquisition, K.Loré.; investigation, O.E., G.J., M.M., K.Lenart., X.Y., R.A.C., and A.R.; methodology, G.J., X.Y., R.A.C., and K.Lenart.; resources, K.Loré.; supervision, K.Loré.; visualization, O.E. and G.J.; writing—original draft, O.E., G.J., A.R., and K.Loré.; writing—review & editing, all authors.

DECLARATION OF INTERESTS

The authors declare no competing interests.

SUPPLEMENTAL INFORMATION

Supplemental information can be found online at https://doi.org/10.1016/j.omtn.2025.102588.

REFERENCES

1. Alberer, M., Gnad-Vogt, U., Hong, H.S., Mehr, K.T., Backert, L., Finak, G., Gottardo, R., Bica, M.A., Garofano, A., Koch, S.D., et al. (2017). Safety and immunogenicity of a mRNA rabies vaccine in healthy adults: an open-label, non-randomised, prospective, first-in-human phase 1 clinical trial. Lancet *390*, 1511–1520.

2. Kremsner, P.G., Ahuad Guerrero, R.A., Arana-Arri, E., Aroca Martinez, G.J., Bonten, M., Chandler, R., Corral, G., De Block, E.J.L., Ecker, L., Gabor, J.J., et al. (2022). Efficacy and safety of the CVnCoV SARS-CoV-2 mRNA vaccine candidate in ten countries in Europe and Latin America (HERALD): a randomised, observer-blinded, placebo-controlled, phase 2b/3 trial. Lancet Infect. Dis. *22*, 329–340.

3. Karikó, K., Buckstein, M., Ni, H., and Weissman, D. (2005). Suppression of RNA recognition by Toll-like receptors: the impact of nucleoside modification and the evolutionary origin of RNA. Immunity *23*, 165–175.

4. Verbeke, R., Hogan, M.J., Loré, K., and Pardi, N. (2022). Innate immune mechanisms of mRNA vaccines. Immunity *55*, 1993–2005.

5. Pardi, N., and Krammer, F. (2024). mRNA vaccines for infectious diseases — advances, challenges and opportunities. Nat. Rev. Drug Discov. *23*, 838–861. https://doi.org/10.1038/s41573-024-01042-y.

6. Thess, A., Grund, S., Mui, B.L., Hope, M.J., Baumhof, P., Fotin-Mleczek, M., and Schlake, T. (2015). Sequence-engineered mRNA Without Chemical Nucleoside Modifications Enables an Effective Protein Therapy in Large Animals. Mol. Ther. *23*, 1456–1464.

7. Lutz, J., Lazzaro, S., Habbeddine, M., Schmidt, K.E., Baumhof, P., Mui, B.L., Tam, Y. K., Madden, T.D., Hope, M.J., Heidenreich, R., and Fotin-Mleczek, M. (2017). Unmodified mRNA in LNPs constitutes a competitive technology for prophylactic vaccines. NPJ Vaccines *2*, 29.

8. Hong, H.S., Koch, S.D., Scheel, B., Gnad-Vogt, U., Schröder, A., Kallen, K.J., Wiegand, V., Backert, L., Kohlbacher, O., Hoerr, I., et al. (2016). Distinct transcriptional changes in non-small cell lung cancer patients with multi-antigenic RNActive® CV9201 immunotherapy. OncoImmunology *5*, e1249560.

9. Kranz, L.M., Diken, M., Haas, H., Kreiter, S., Loquai, C., Reuter, K.C., Meng, M., Fritz, D., Vascotto, F., Hefesha, H., et al. (2016). Systemic RNA delivery to dendritic cells exploits antiviral defence for cancer immunotherapy. Nature *534*, 396–401.

10. Gainor, J.F., Patel, M.R., Weber, J.S., Gutierrez, M., Bauman, J.E., Clarke, J.M., Julian, R., Scott, A.J., Geiger, J.L., Kirtane, K., et al. (2024). T Cell Responses to Individualized Neoantigen Therapy mRNA-4157 (V940) Alone or in Combination With Pembrolizumab in the Phase 1 KEYNOTE-603 Study. Cancer Discov. *14*, 2209–2223. https://doi.org/10.1158/2159-8290.CD-24-0158.

11. Weber, J.S., Carlino, M.S., Khattak, A., Meniawy, T., Ansstas, G., Taylor, M.H., Kim, K.B., McKean, M., Long, G.V., Sullivan, R.J., et al. (2024). Individualised neoantigen therapy mRNA-4157 (V940) plus pembrolizumab versus pembrolizumab monotherapy in resected melanoma (KEYNOTE-942): a randomised, phase 2b study. Lancet *403*, 632–644.

12. Sayour, E.J., Boczkowski, D., Mitchell, D.A., and Nair, S.K. (2024). Cancer mRNA vaccines: clinical advances and future opportunities. Nat. Rev. Clin. Oncol. *21*, 489–500.

13. Blasi, M., Wescott, E.C., Baker, E.J., Mildenberg, B., LaBranche, C., Rountree, W., Haynes, B.F., Saunders, K.O., Moody, M.A., Negri, D., et al. (2020). Therapeutic vaccination with IDLV-SIV-Gag results in durable viremia control in chronically SHIV-infected macaques. NPJ Vaccines *5*, 36.

14. Valentin, A., Bergamaschi, C., Rosati, M., Angel, M., Burns, R., Agarwal, M., Gergen, J., Petsch, B., Oostvogels, L., Loeliger, E., et al. (2022). Comparative immunogenicity of an mRNA/LNP and a DNA vaccine targeting HIV gag conserved elements in macaques. Front. Immunol. *13*, 945706.

15. Yan, X., Arcoverde Cerveira, R., Ols, S., Lenart, K., Hellgren, F., Miranda, M., Engstrand, O., Reinhardt, A., Eriksson, B., and Loré, K. (2025). Biochemical and hematological reference intervals in rhesus and cynomolgus macaques and implications for vaccine and drug development. Lab. Anim. *54*, 141–155. https://doi.org/10.1038/s41684-025-01547-y.

16. Tahtinen, S., Tong, A.-J., Himmels, P., Oh, J., Paler-Martinez, A., Kim, L., Wichner, S., Oei, Y., McCarron, M.J., Freund, E.C., et al. (2022). IL-1 and IL-1ra are key regulators of the inflammatory response to RNA vaccines. Nat. Immunol. *23*, 532–542.

17. Hellgren, F., Rosdahl, A., Arcoverde Cerveira, R., Lenart, K., Ols, S., Gwon, Y.D., Kurt, S., Delis, A.M., Joas, G., Evander, M., et al. (2024). Modulation of innate immune response to mRNA vaccination after SARS-CoV-2 infection or sequential vaccination in humans. JCI Insight *9*, e175401.

18. Lenart, K., Hellgren, F., Ols, S., Yan, X., Cagigi, A., Cerveira, R.A., Winge, I., Hanczak, J., Mueller, S.O., Jasny, E., et al. (2022). A third dose of the unmodified COVID-19 mRNA vaccine CVnCoV enhances quality and quantity of immune responses. Mol. Ther. Methods Clin. Dev. *27*, 309–323.

19. Hellgren, F., Cagigi, A., Arcoverde Cerveira, R., Ols, S., Kern, T., Lin, A., Eriksson, B., Dodds, M.G., Jasny, E., Schwendt, K., et al. (2023). Unmodified rabies mRNA vaccine elicits high cross-neutralizing antibody titers and diverse B cell memory responses. Nat. Commun. *14*, 3713.

20. Arunachalam, P.S., Scott, M.K.D., Hagan, T., Li, C., Feng, Y., Wimmers, F., Grigoryan, L., Trisal, M., Edara, V.V., Lai, L., et al. (2021). Systems vaccinology of the BNT162b2 mRNA vaccine in humans. Nature *596*, 410–416.

21. Liang, F., Lindgren, G., Lin, A., Thompson, E.A., Ols, S., Röhss, J., John, S., Hassett, K., Yuzhakov, O., Bahl, K., et al. (2017). Efficient Targeting and Activation of Antigen-Presenting Cells In Vivo after Modified mRNA Vaccine Administration in Rhesus Macaques. Mol. Ther. *25*, 2635–2647.

22. Li, S., Rouphael, N., Duraisingham, S., Romero-Steiner, S., Presnell, S., Davis, C., Schmidt, D.S., Johnson, S.E., Milton, A., Rajam, G., et al. (2014). Molecular signatures of antibody responses derived from a systems biology study of five human vaccines. Nat. Immunol. *15*, 195–204.

23. Agrawal, A., Balcı, H., Hanspers, K., Coort, S.L., Martens, M., Slenter, D.N., Ehrhart, F., Digles, D., Waagmeester, A., Wassink, I., et al. (2024). WikiPathways 2024: next generation pathway database. Nucleic Acids Res. *52*, D679–D689.

24. Anderson, E.J., Rouphael, N.G., Widge, A.T., Jackson, L.A., Roberts, P.C., Makhene, M., Chappell, J.D., Denison, M.R., Stevens, L.J., Pruijssers, A.J., et al. (2020). Safety and Immunogenicity of SARS-CoV-2 mRNA-1273 Vaccine in Older Adults. N. Engl. J. Med. *383*, 2427–2438.

25. Maruyama, A., Sawa, T., Teramukai, S., and Katoh, N. (2022). Adverse reactions to the first and second doses of Pfizer-BioNTech COVID-19 vaccine among healthcare workers. J. Infect. Chemother. *28*, 934–942.

26. Li, C., Lee, A., Grigoryan, L., Arunachalam, P.S., Scott, M.K.D., Trisal, M., Wimmers, F., Sanyal, M., Weidenbacher, P.A., Feng, Y., et al. (2022). Mechanisms of innate and adaptive immunity to the Pfizer-BioNTech BNT162b2 vaccine. Nat. Immunol. *23*, 543–555.

27. Huang, J., Long, Z., Jia, R., Wang, M., Zhu, D., Liu, M., Chen, S., Zhao, X., Yang, Q., Wu, Y., et al. (2021). The Broad Immunomodulatory Effects of IL-7 and Its Application In Vaccines. Front. Immunol. *12*, 680442.

28. Pachynski, R.K., Morishima, C., Szmulewitz, R., Harshman, L., Appleman, L., Monk, P., Bitting, R.L., Kucuk, O., Millard, F., Seigne, J.D., et al. (2021). IL-7 expands lymphocyte populations and enhances immune responses to sipuleucel-T in patients with metastatic castration-resistant prostate cancer (mCRPC). J. Immunother. Cancer *9*, e002903.

29. Zhao, Y., Wei, K., Chi, H., Xia, Z., and Li, X. (2022). IL-7: A promising adjuvant ensuring effective T cell responses and memory in combination with cancer vaccines? Front. Immunol. *13*, 1022808.

30. Alameh, M.G., Tombácz, I., Bettini, E., Lederer, K., Sittplangkoon, C., Wilmore, J.R., Gaudette, B.T., Soliman, O.Y., Pine, M., Hicks, P., et al. (2021). Lipid nanoparticles enhance the efficacy of mRNA and protein subunit vaccines by inducing robust T follicular helper cell and humoral responses. Immunity *54*, 2877–2892.e7.

31. Netea, M.G., Domínguez-Andrés, J., Barreiro, L.B., Chavakis, T., Divangahi, M., Fuchs, E., Joosten, L.A.B., van der Meer, J.W.M., Mhlanga, M.M., Mulder, W.J.M., et al. (2020). Defining trained immunity and its role in health and disease. Nat. Rev. Immunol. *20*, 375–388.

32. Stevens, N.E., Ryan, F.J., Messina, N.L., Blake, S.J., Norton, T.S., Germano, S., James, J., Eden, G.L., Tee, Y.C., Lynn, M.A., et al. (2023). No evidence of durable trained immunity after two doses of adenovirus-vectored or mRNA COVID-19 vaccines. J. Clin. Investig. *133*, e171742.

33. Oda, Y., Kumagai, Y., Kanai, M., Iwama, Y., Okura, I., Minamida, T., Yagi, Y., Kurosawa, T., Chivukula, P., Zhang, Y., and Walson, J.L. (2024). 12-month persistence of immune responses to self-amplifying mRNA COVID-19 vaccines: ARCT-154 versus BNT162b2 vaccine. Lancet Infect. Dis. *24*, e729–e731.

34. Hồ, N.T., Hughes, S.G., Ta, V.T., Phan, L.T., Đỗ, Q., Nguyễn, T.V., Phạm, A.T.V., Thị Ngọc Đặng, M., Nguyễn, L.V., Trịnh, Q.V., et al. (2024). Safety, immunogenicity and efficacy of the self-amplifying mRNA ARCT-154 COVID-19 vaccine: pooled phase 1, 2, 3a and 3b randomized, controlled trials. Nat. Commun. *15*, 4081.

35. Medina-Magües, L.G., Gergen, J., Jasny, E., Petsch, B., Lopera-Madrid, J., Medina-Magües, E.S., Salas-Quinchucua, C., and Osorio, J.E. (2021). mRNA Vaccine Protects against Zika Virus. Vaccines (Basel). *9*, 1464.

36. Gebre, M.S., Rauch, S., Roth, N., Yu, J., Chandrashekar, A., Mercado, N.B., He, X., Liu, J., McMahan, K., Martinot, A., et al. (2022). Optimization of non-coding regions for a non-modified mRNA COVID-19 vaccine. Nature *601*, 410–414.

37. Petsch, B., Schnee, M., Vogel, A.B., Lange, E., Hoffmann, B., Voss, D., Schlake, T., Thess, A., Kallen, K.-J., Stitz, L., and Kramps, T. (2012). Protective efficacy of in vitro synthesized, specific mRNA vaccines against influenza A virus infection. Nat. Biotechnol. *30*, 1210–1216.

38. Lenart, K., Arcoverde Cerveira, R., Hellgren, F., Ols, S., Sheward, D.J., Kim, C., Cagigi, A., Gagne, M., Davis, B., Germosen, D., et al. (2024). Three immunizations with Novavax's protein vaccines increase antibody breadth and provide durable protection from SARS-CoV-2. NPJ Vaccines *9*, 17.

39. Ewels, P.A., Peltzer, A., Fillinger, S., Patel, H., Alneberg, J., Wilm, A., Garcia, M.U., Di Tommaso, P., and Nahnsen, S. (2020). The nf-core framework for community-curated bioinformatics pipelines. Nat. Biotechnol. *38*, 276–278.

40. Soneson, C., Love, M.I., and Robinson, M.D. (2015). Differential analyses for RNA-seq: transcript-level estimates improve gene-level inferences. F1000Res. *4*, 1521.

41. Rainer, J., Gatto, L., and Weichenberger, C.X. (2019). ensembldb: an R package to create and use Ensembl-based annotation resources. Bioinformatics *35*, 3151–3153.

42. Love, M.I., Huber, W., and Anders, S. (2014). Moderated estimation of fold change and dispersion for RNA-seq data with DESeq2. Genome Biol. *15*, 550.

43. Yu, G., Wang, L.G., Han, Y., and He, Q.Y. (2012). clusterProfiler: an R package for comparing biological themes among gene clusters. OMICS *16*, 284–287.

44. Yan, X., Ols, S., Arcoverde Cerveira, R., Lenart, K., Hellgren, F., Ye, K., Cagigi, A., Buggert, M., Nimmerjahn, F., Falkesgaard Højen, J., et al. (2023). Cell targeting and immunostimulatory properties of a novel Fcγ-receptor-independent agonistic anti-CD40 antibody in rhesus macaques. Cell. Mol. Life Sci. *80*, 189.

OMTN, Volume *36*

Supplemental information

Immunogenicity and innate immunity to high-dose and repeated vaccination of modified mRNA versus unmodified mRNA

Olivia Engstrand, Gustav Joas, Marcos C. Miranda, Xianglei Yan, Klara Lenart, Rodrigo Arcoverde Cerveira, Annika Reinhardt, and Karin Loré

SUPPLEMENTAL MATERIALS

SUPPLEMENTAL FIGURES

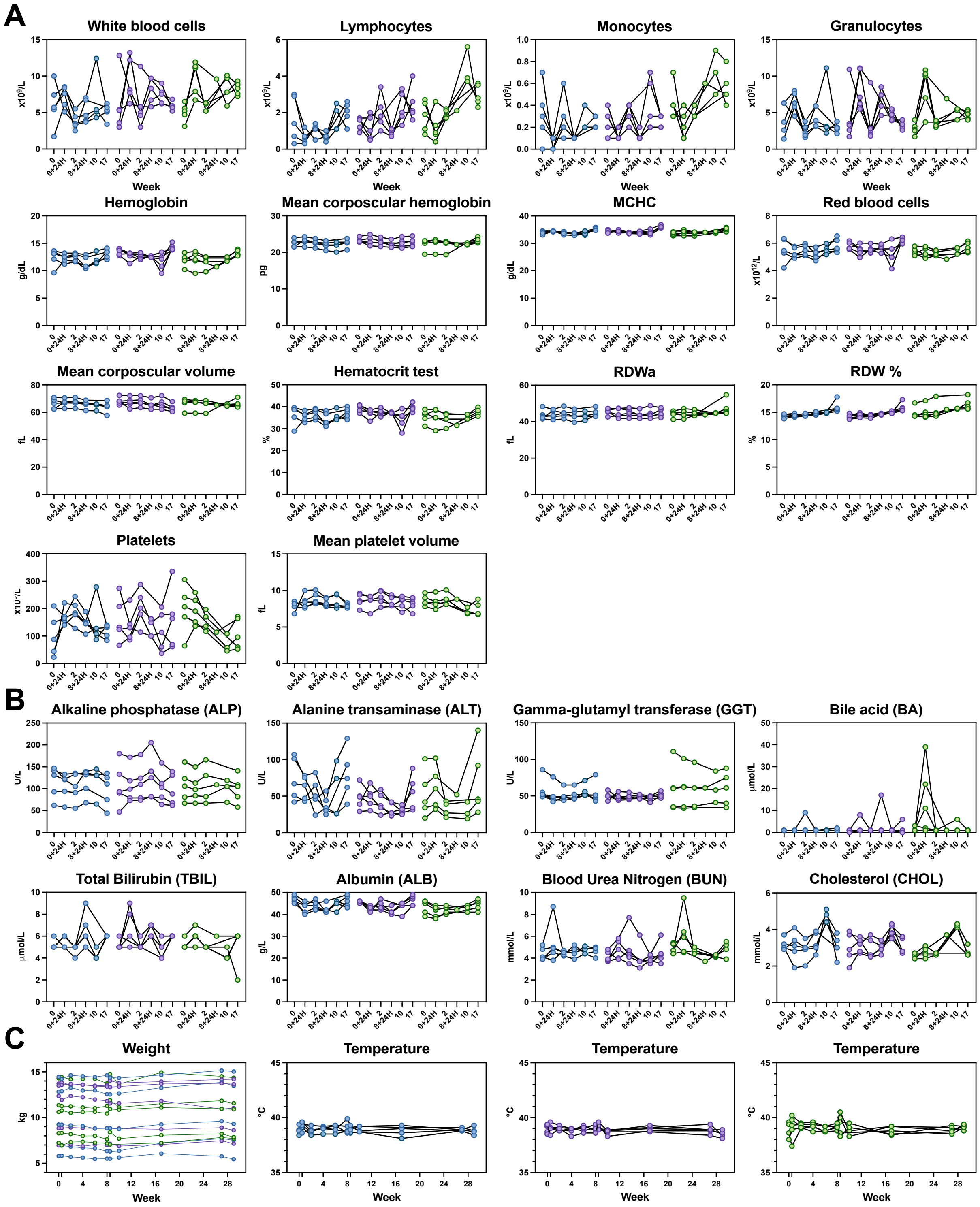

Figure S1: Safety data after repetitive immunizations. Unmodified mRNA 160µg (blue), Modified mRNA 400µg (purple) and Modified mRNA 800µg (green). (A) Hematological paramaters and (B) Clinical chemistry at baseline, 24 hours and 2 weeks post first immunization and 24 hours, 2 weeks and 9 weeks post fifth immunization. (C) Weight and temperature at each sampling time point.

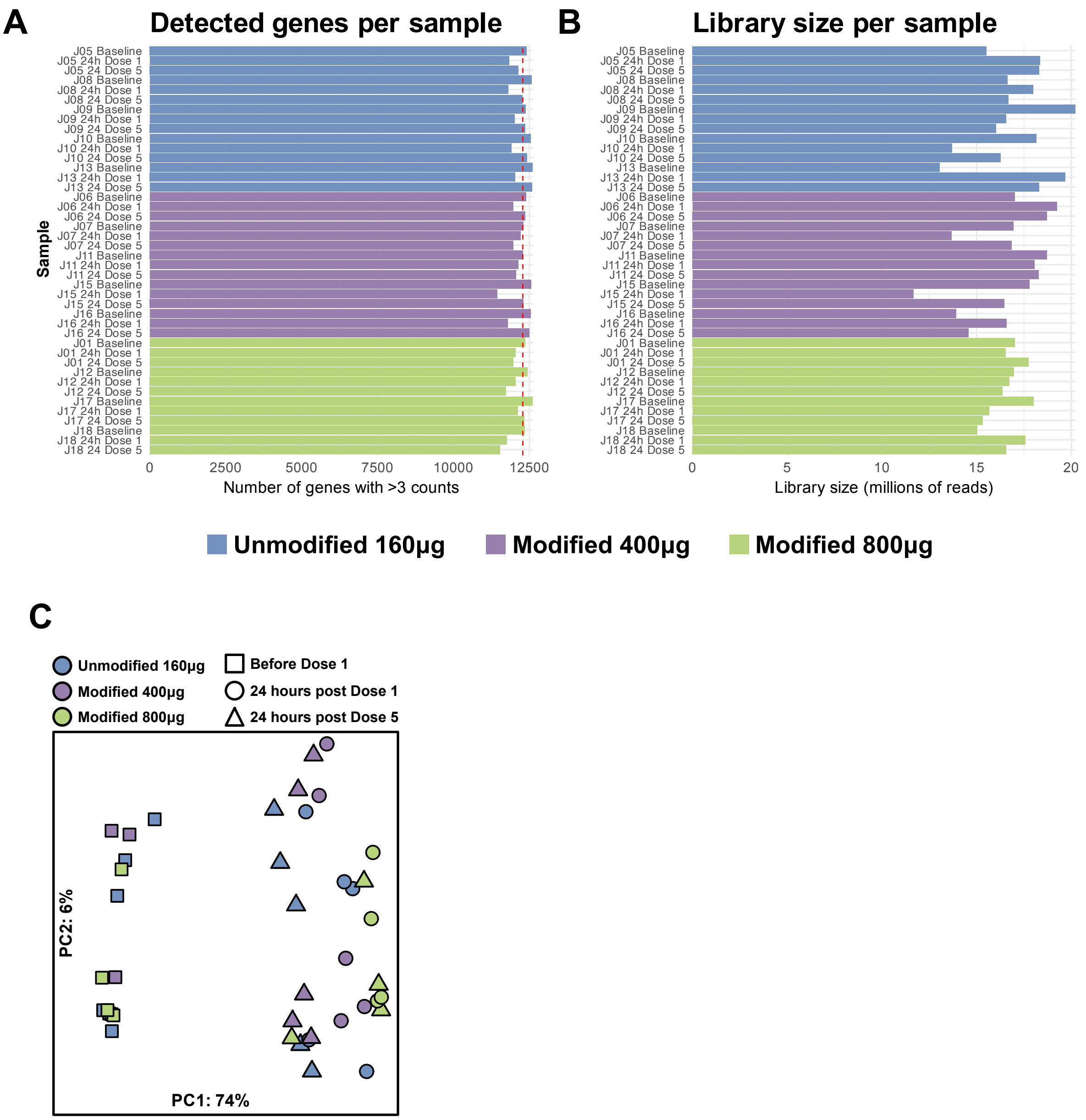

Figure S2: RNA sequencing quality control (A) Bars show the number of genes with >3 raw counts in each RNA-seq sample. The dashed red line indicates the median number of detected genes across all samples. (B) Total read counts (in millions) for each sample prior to normalization. (C) Principal Component Analysis colored by group and shaped by sampling time point.

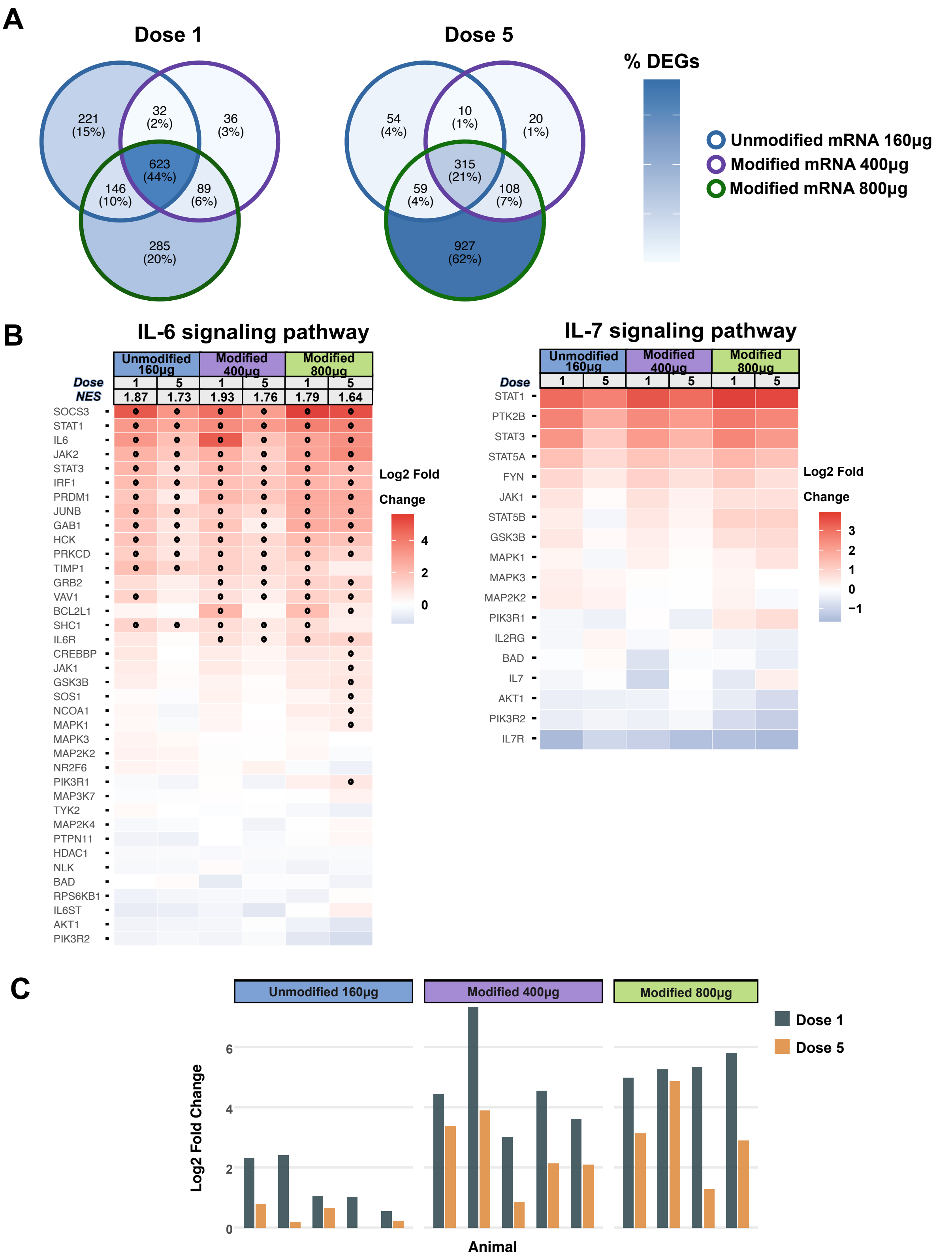

Figure S3: Transcriptomic Changes Across Vaccine Groups and Timepoints (A) Venn diagrams illustrating the percentage of overlapping DEGs across groups and doses. Exclusive regions represent DEGs unique to each group. Shared regions, including the central intersection, show DEGs common to two or more groups. (B) Heatmaps of $\log_2$ fold changes ($\log_2$FC) for genes in the IL-6 and IL-7 signaling pathways, as defined in the WikiPathways database, across groups and timepoints. Black circles indicate core enriched genes as identified by Gene Set Enrichment Analysis. (C) Barplot showing *gag* expression levels detected by RNA-seq 24 hours after vaccination, stratified by vaccine group and dose. gag was undetectable at baseline. The negative control gene, env (not shown), was not detected in any sample. NES = normalized enrichment score.

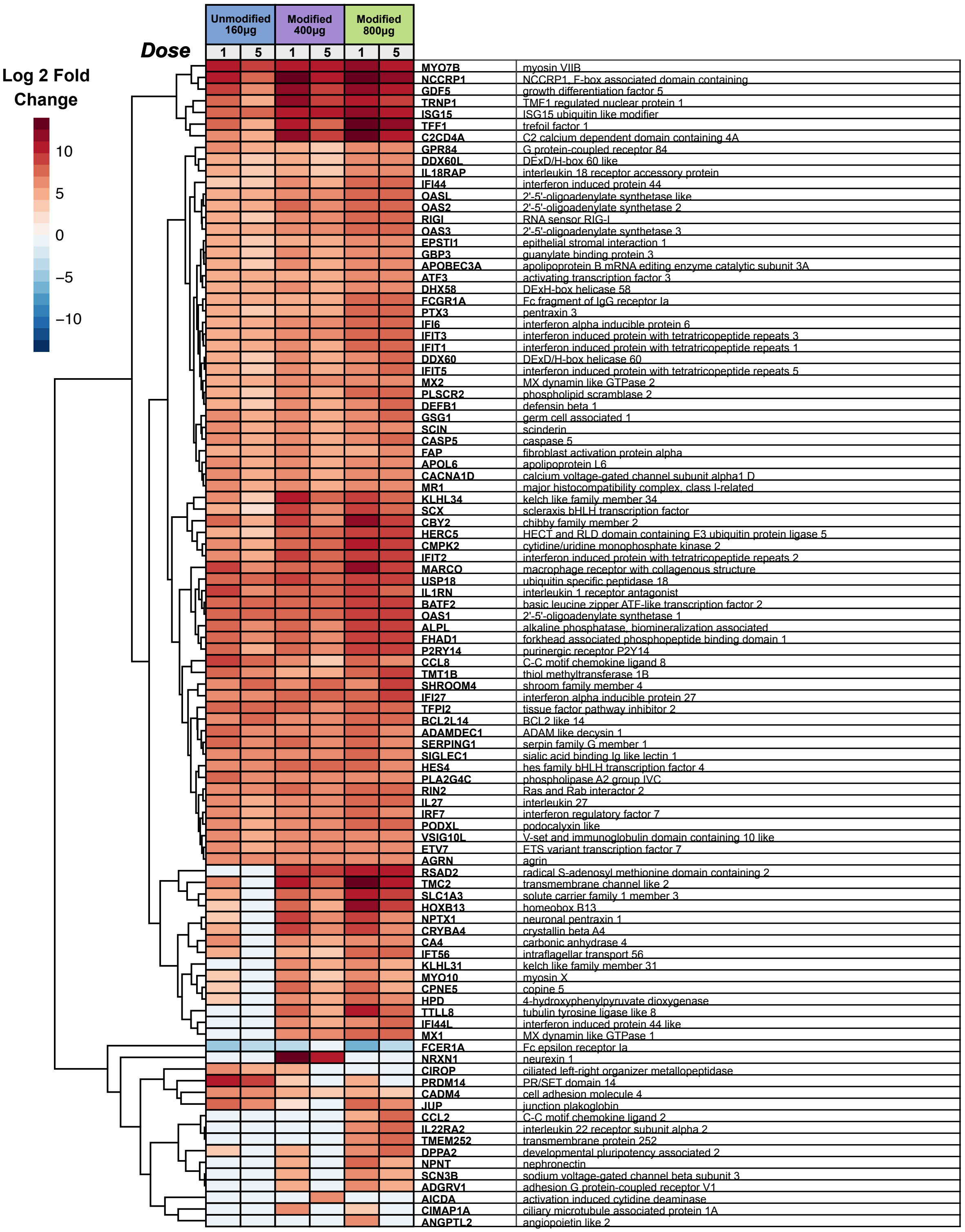

Figure S4: Top 100 differentially expressed genes (DEGs) across groups and timepoints following vaccination. DEGs with the highest absolute $\log_2$ fold changes and False Discovery Rate adjusted *p*-values < 0.05 across all groups and timepoints. Comparing baseline (0 hours, pre-immunization) to 24 hours post-immunization following the first and fifth doses. Gene descriptions are shown on the right. Genes were hierarchically clustered using Ward's method. Missing values were imputed as 0.

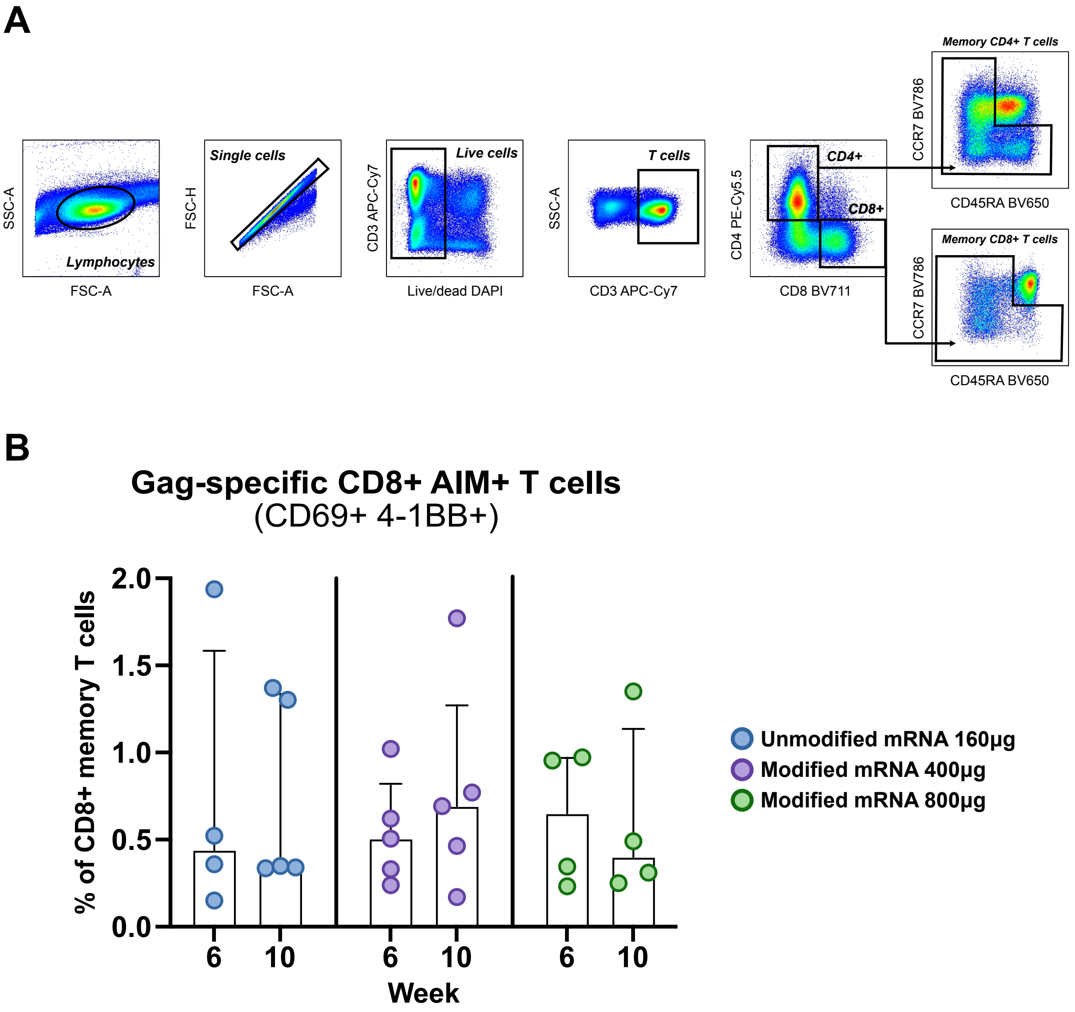

Figure S5: Gag-specific T cell responses. (A) Representative gating strategy to identify memory T cell subsets. (B) Frequency of Gag-specific CD8+ CD69+ 4-1BB+ T cells analyzed from antigen recall assay with fresh PBMCs. Graph is showing background-subtracted frequencies calculated based on corresponding DMSO-control, Data presented as group medians (IQR).

SUPPLEMENTAL TABLES

Table S1: Immunophenotyping antibody panel.

Fluorochrome	Marker	Clone	Company	Cat. no.
FITC	CD40	5C3	BioLegend	334306
PE	NKg2a (CD159a)	Z199	Beckman Coulter	IM3291U
BV421	CD80	L307.4	BD	564160
PE-Dazzle 594	CCR7	G043H7	Biolegend	353236
PerCp-Cy5.5	CD123	7G3	BD	558714
APC-Cy7	CD3	SP34-2	BD	557757
APC	CD66	TET2	Miltenyi	130-118-539
BV786	CD70	Ki-24	BD	565338
BV650	HLA-DR	L243	Biolegend	307650
PE-Cy7	CD11c	3.9	BioLegend	301608
AF700	CD16	38G	BD	560713
BV605	CD20	2H7	BioLegend	302334
BV510	CD14	M5E2	Biolegend	301842
DAPI (AF350)	Live Dead Fixable Blue	-	Life Tech	L-23105

Table S2: T cell assay antibody panel

Fluorochrome	Marker	Clone	Company	Cat. no.
CD107a	BV421	H4A3	Biolegend	328626
OX40	BV510	L106	BD	745040
CCR7	BV786	G043H7	Biolegend	353230
CD8a	BV711	RPA-T8	Biolegend	301044
CD4	PE-Cy55	S3.5	Invitrogen	MHCD0418
CD45RA	BV650	5H9	BD	740608
4-1BB	APC	4B4-1	BD	550890
IL-2	BV605	MQ1-17H12•	BD	564165
CD69	ECD	TP1.55.3	Beckman	6607110
CD3	APC-Cy7	SP34.2	BD	557757
IFNg	AF700	B27	Biolegend	506516
DAPI (AF350)	Live Dead Fixable Blue	-	Life Tech	L-23105
